# Effective investment in women's futures: Schooling with learning

**DOI:** 10.1016/j.ijedudev.2021.102464

**Published:** 2021-10

**Authors:** Michelle Kaffenberger, Lant Pritchett

**Affiliations:** Blavatnik School of Government, University of Oxford

**Keywords:** Returns to schooling, Education, Literacy, Child mortality, Fertility, Empowerment, Non-pecuniary outcomes

## Abstract

Women's schooling has long been regarded as one of the best investments in development. Yet countries vary widely in the extent to which a year of schooling conveys useful skills and knowledge and hence schooling and education cannot be treated as synonyms. Using two different cross-nationally comparable data sets which contain measures of schooling, assessments of literacy, and life outcomes for more than 50 countries we show the association of women's *education*, defined as schooling and the acquisition of literacy, with four life outcomes (fertility, child mortality, empowerment, and financial practices) is much larger than the standard estimates of the gains from schooling alone. In our preferred instrumental variables (IV) estimates, that correct for the attenuation bias induced by measurement error, the gain from *education* is more than 3 times larger than the standard OLS estimates of gains from schooling alone. While our results are not causal estimates, if anything like our results are true in the causal pathways whereby schooling and learning lead to improvements in women's well-being this implies the estimates of the costs and benefits of expansions in grade attainment versus improving learning need to be adjusted accordingly.

## Highlights

• Most analysis of the association of girl's education with life outcomes uses measures only of school grade attainment, not learning.
• Data for more than 50 countries are used to analyze associations between schooling, literacy, and fertility, child mortality, women's empowerment, and financial behaviors.
• The associations of women's education (primary schooling and basic literacy) are much larger than standard methods using only schooling data would suggest.
• Disentangling the role of schooling and learning is important for informing investments in women's futures.

## 1 Motivation and introduction^1^

It has long been acknowledged that women's schooling is an investment with high pecuniary and non-pecuniary returns. The estimates of the wage return to female schooling are typically as high, or higher, than for males (Montenegro and Patrinos, 2014). Moreover, the widespread availability of household survey data sets (many national and many with at least some cross-national comparability, e.g. DHS, MICS, LSMS, World Values Surveys, Young Lives) with measures of schooling completed and non-pecuniary outcomes (fertility, child mortality, political participation, attitudes, values, health care usage, child nutrition, child school attendance, etc.) has led to thousands of studies comparing life outcomes by individuals’ levels of completed schooling. Oft-cited estimates suggest that child mortality declines 7–9% per year of women's schooling (e.g. Cleland and Van Ginneken, 1988, Cochrane, 1980, United Nations, 1985). An analysis using 915 data sources from 219 countries claimed that female schooling prevented 4.2 million child (under 5) deaths between 1970 and 2000 (Gakidou et al., 2010). Similarly, observational studies have linked years of schooling to reductions in fertility via various pathways such as family size preference, age at first marriage, and contraceptive use (Martin, 1995), and to lower child malnutrition (Keats et al., 2017).

In this enormous literature “schooling” and “education” are generally treated as synonyms. Studies claim to examine the impact^2^ of “education” on pecuniary and non-pecuniary outcomes (e.g. wages, economic growth, women's empowerment, child health, political participation) but actually only examine the empirical relationships of these outcomes with measures of schooling completed. Two recent systematic reviews examining causal links between female “education” and maternal and child health (Mensch et al., 2019) and sexual and reproductive practices (Psaki et al., 2019) included only one study (total, across both reviews) that included any measure of learning, all other relied only on measures of years of levels of schooling completed (both reviews acknowledge this shortcoming of the current literature).

**Fig. 1:**
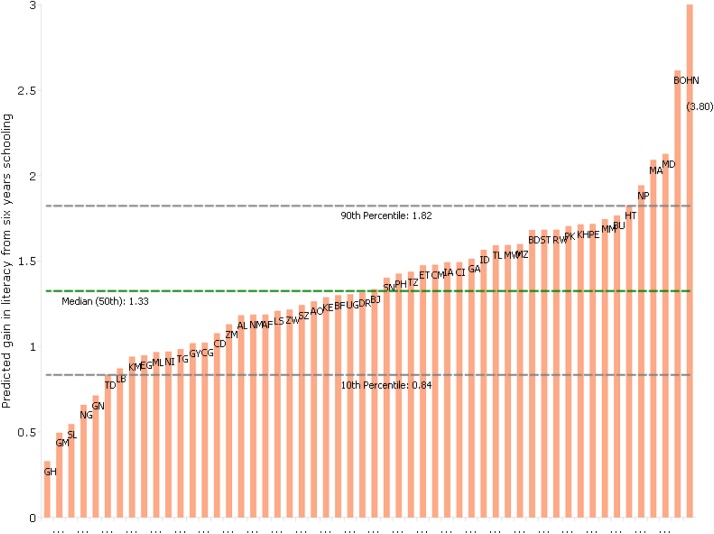
The predicted gain in literacy from six years of schooling varies by an order of magnitude across countries. Source: Authors’ analysis of DHS micro-data. Estimated coefficients are unrestricted, and so the highest coefficients are above the bounds of the 0,1,2 literacy measure.

If years of schooling completed and education (schooling plus learning) were tightly associated within and across countries, conflating the two might be benign. Unfortunately, often “schooling ain’t learning” (Pritchett, 2013) and an increasing number of cross-national comparisons of learning show massive differences in the skills/competencies/capabilities acquired per year of schooling. Fig. 1 is based on the Demographic and Health Survey (DHS) estimates of literacy, which is zero if a woman could not read a sentence, 1 if the woman could read a sentence with some help^3^ and 2 if she could read the sentence with no help. It shows that the average score for a woman with six years of schooling varies from less than 1 in countries like Ghana (GH) and Nigeria (NG)–implying the “typical” woman cannot read at all–to more than 2 implying most women can read a simple sentence.

**Fig. 2:**
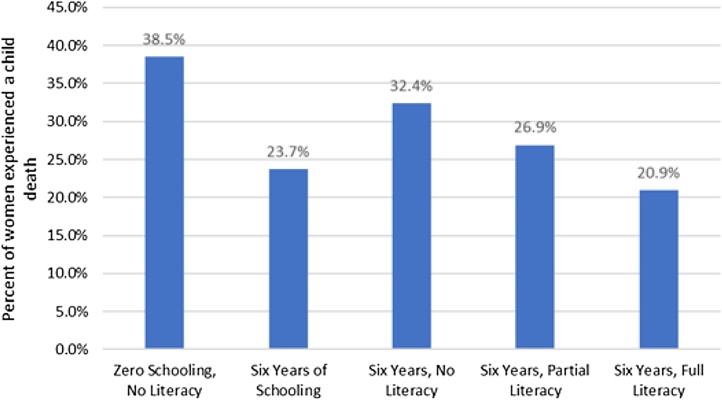
Fraction of women aged 15–49 who have experienced the death of a child, by schooling and literacy levels. Source: Authors’ analysis of DHS micro-data, including N=854,766 women who have ever given birth, from 54 countries.

This generally weak and widely varying connection between schooling and learning implies the impact of *education*, which implies schooling and learning, cannot be conflated with any estimate of the impact of schooling, even if that estimate is causal. Fig. 2 is the cross-tabulation of child mortality (whether, among women who have ever had a child, a woman has ever experienced the death of a child) by the woman's level of schooling and by the DHS measure of literacy using the data from 54 countries. Of women with no schooling and no literacy (unschooled and uneducated) 38.5% have experienced a child death. Among women with six years of schooling complete but who could not read the sentence at all (schooled, but not educated) 32.4% had experienced a child death, only 6.1 percentage points lower than women without either schooling or literacy. Among women with six years of schooling complete and who could read a sentence without help, which we call basic education, defined as both completion of primary schooling plus acquiring basic literacy, only 20.9% had experienced a child death, 17.6% percentage points lower than women with no formal schooling or literacy. The difference in child mortality between women with six years of schooling complete with and without reading is almost twice as big (32.4%–20.9%=11.5%) as the gap between women with no schooling who cannot read and those with six years of schooling and cannot read (38.5%–32.4%=6.1%). The existing literature that compares outcomes with and without schooling (and this is true of cross-tabulations, regressions, or causal estimates) produces estimates of the impact of schooling that are some weighted average of the gains from schooling for women who achieved very different levels of learning from their schooling. To the extent that learning is any part of the causal pathway whereby schooling leads to better outcomes this implies estimates of schooling alone will understate the gains from education (schooling with learning).

We use DHS data from 54 countries (and a total of 128 survey rounds) and Financial Inclusion Insights (FII) data from 10 countries to estimate the empirical associations between schooling (years completed) and a measure of learning (ability to read) with four non-pecuniary adult outcomes: fertility, child mortality, and an index of women's empowerment (DHS) and an index of financial behaviors (FII). Using two separate data sources, with two different literacy tests, administered to two different subsets of national populations (women of child bearing ages only in the DHS versus all adults for the FII), across four life outcome variables across many countries produces remarkably consistent empirical results.

These empirical findings are all relevant to policy decisions, as optimal allocation of effort (or funding) to increase years of schooling versus to improve learning per year necessarily depends on the relative costs and the relative life outcome benefits. While there is increasing evidence about the cost effectiveness of various “interventions” in increasing either schooling or learning per year of schooling, they are insufficient for informing policy without consideration of the impacts on life outcomes of each, as either simplistic assumption that all the benefits are accomplished just by time served in schooling, or that all of the benefits are completely captured by learning metrics, are likely to be false.

## 2 Equations; data on schooling, literacy, and outcomes; and methods

### 2.1 Equations

Suppose a life outcome (Y) for a specific woman (i) living in country c and locality j is a linear function of her years of schooling completed (S), her extent of learning (L), and other factors about the woman that are in the data (Z), e.g. her age, whether she lives in a urban or rural location, a household wealth index) plus everything else that affects outcomes besides S,L,Z:

(1) $$Y^{i,c,j}=\alpha^{c}+\beta_{S|L,Z}^{c}*S_{i,j}+\beta_{L|S,Z}^{c}*L_{i,j}+\theta_{Z|L,S}^{c}*Z_{i,j}+\mathrm{everything}\mathrm{else}$$

Also suppose the learning achieved by the $i^{\mathrm{th}}$ woman is linked to her years of schooling in country *c* and locality *j* by a simple linear equation (Eq. (2)), where $\gamma^{c,j}$ is the learning produced by a year of schooling, plus all else that affects her learning in an error term ϵ:

(2) $$L^{i,c,j}=\eta^{c}+\gamma^{c,j}*S_{i,c,j}+\epsilon$$

These very restrictive assumptions have the benefit of a simple notation to clarify concepts.

First, $\beta_{S|L,Z}^{c}$, from Eq. (1) is the partial derivative of the life outcome with respect to schooling, which holds the extent of learning and the Zs fixed. This is the “partial” or “direct” impact, the impact of spending time in school in and of itself, holding all else–including learning–constant.

Second, the *total*^4^ derivative of life outcomes w.r.t. schooling in Eq. (3) is the sum of the partial (or “direct”) impact of schooling ($\beta_{S|L,Z}^{c}$) and the pathway whereby schooling raises learning ($\gamma^{c,j}$ from Eq. (2)) and this increased learning affects outcomes ($\beta_{L|S,Z}^{c}$).

(3) $$\mathrm{Total}\mathrm{impact}\mathrm{of}\mathrm{schooling}=\beta_{S|L,Z}^{c}+\gamma^{c,j}*\beta_{L|S,Z}^{c}$$

If primary schooling consists of S years of schooling at the level of learning per year of schooling ($\gamma^{c,j}$) then the total impact of primary schooling on outcome Y is:

(4) $$\mathrm{Total}\mathrm{impact}\mathrm{of}\mathrm{primary}\mathrm{schooling}(Z\;\mathrm{fixed}):\Delta Y=\beta_{S|L,Z}^{c}*\Delta S+\beta_{L|S,Z}^{c}*(\gamma^{c,j})*\Delta S$$

We call the “impact of education” the direct impact of primary schooling *plus* the impact of a *defined* level of basic learning, ΔL:

(5) $$\mathrm{Impact}\mathrm{of}\mathrm{basic}\mathrm{education}:\Delta Y=\beta_{S|L,Z}^{c}*\Delta S+\beta_{L|S,Z}^{c}*\Delta L$$

These simple equations have three substantive implications.

First, if any of the causal pathway from schooling to life outcomes is through learning, then *all* estimates of the impact of women's primary *schooling* on life outcomes *underestimate* the impact of *basic education* on life outcomes. Furthermore, as seen in Eq. (6), the magnitude of the difference depends on: (a) the extent to which the actual learning from primary school falls short of the learning level that defines basic education ($\Delta S*\gamma^{c}-\Delta L$) and (b) the extent to which learning affects life outcomes, conditional on schooling ($\beta_{L|S,Z}^{c}$).^5^

(6) $$\mathrm{Difference}\mathrm{between}\mathrm{impact}\mathrm{of}\mathrm{primary}\mathrm{schooling}\mathrm{and}\mathrm{impact}\mathrm{of}\mathrm{basic}\mathrm{education}=\beta_{L|S,Z}^{c}*(\gamma^{c}*\Delta S-\Delta L)$$

Second, a consequence of Eq. (6) and Fig. 1 is that since $\gamma^{c}$ differs widely across countries, the impact of schooling (Eq. (4)) will differ across countries. Take two countries, A and B. Even if the “direct” impact of schooling ($\beta_{S|L,Z}$) and the “direct” impact of learning ($\beta_{L|S,Z}$) and hence the impact of education (Eq. (5)) are the same in country A and country B, to the extent that $\gamma^{c}$ (learning per year of schooling) differs, if learning matters for outcomes ($\beta_{L|S,Z}>0$), then the total impact of schooling (Eq. (4)) would differ. If, for example, all of the impact on outcomes of schooling was through learning ($\beta_{S|L,Z}=0$) and countries A and B had the same impact on outcomes of learning ($\beta_{L|S,Z}^{A}=\beta_{L|S,Z}^{B}$), then the impact of schooling in country A versus country B would just be the ratio $\gamma_{A}^{c}/\gamma_{B}^{c}$. Using the results in Fig. 1 this would imply the 90th percentile learning country would have an estimated impact of schooling of 2.16 (=1.82/.84) times higher than the 10th percentile learning country if all impact were through learning. This implies there cannot be “external validity” of estimates of the impact of schooling across contexts/countries as even unbiased estimates of the LATE of an additional year of schooling for country A and country B will have to differ if learning matters at all ($\beta_{L|S,Z}>0$) because the actual LATE depends on learning and hence differs between A and B.^6^

Third, a point we return to in Section 4 below with empirical estimates in hand, the efficacy of various possible investments in improving women's life outcomes through education depends on the empirical magnitudes of the causal pathways of schooling and learning in improving outcomes. For instance, Barrera-Osorio et al. (2018) evaluated two different scholarships given to fourth grade students in Cambodia, one merit based and one needs based, which were awarded in 2008. In their long-term follow-up, nine years after the scholarship began, they found that while both programs had roughly equal effects on additional schooling, only the merit-based scholarship had any impact on learning or on any other measured life outcome. An evaluation of these alternative scholarship designs solely on the basis of additional S would have regarded them as equally cost effective in units of S gained per dollar. But a fuller analysis tracing through to learning and to outcomes revealed one design (“merit-based”) produced more S *and* more L and led to impact on outcomes whereas the other design (“need-based”) produced only more S but not more L (hence less than would have been expected from the additional schooling) and had no demonstrable impact on life outcomes and hence was massively less cost-effective at producing improved outcomes. This is not an argument for merit-based scholarships, but rather that children may need improvements in schooling *and* learning to experience improvements in life outcomes, and that these cannot be assumed to result just from more years of schooling. Since the total impact of schooling on life outcomes depends on both the years of schooling completed and on the learning acquired from that schooling these are alternative potential priorities for spending and the optimal mix depends on the actual magnitudes.

### 2.2 DHS and FII data on schooling and literacy

In order to estimate the equations above we used household survey data across large numbers of countries that include: a measure of schooling, life outcome variables, individual and household level co-variates, and, most importantly, an enumerator-administered literacy test. This section describes each of those for our two data sources, DHS and FII. By using a large number of countries, multiple life outcome indicators, and two completely different data sources we are confident our results are not an artefact of any particular quirk of country or data.

The DHS and FII are nationally representative sample household surveys which use a common questionnaire and each produce comparable data across multiple developing countries. We use the 128 DHS survey rounds from 54 countries which contain the literacy assessment introduced around 2000. The DHS survey chooses one woman aged 15 to 49 (reproductive age) from each sampled household to complete a detailed women's questionnaire, which contains the literacy assessment.

The DHS survey instrument asks each sampled woman whether she attended school and if so, the highest level she attended (primary, secondary, or tertiary), and also asks the highest grade she attended within the reported level. We use this self-reported highest grade attained as our measure of schooling.

The DHS literacy assessment is administered only to women who report completing primary school or less as their highest level of schooling (and this unavoidably complicates considerations of how selectivity affects the estimates of impact, discussed below). Enumerators are provided with cards in the variety of languages they expect to encounter and each woman is asked to read a single sentence in any language she chooses. Hence this not an assessment of literacy in English or even the dominant national language but of a woman's ability to read in *any* language.^7^ The cards contain one simple sentence in the woman's selected language, like:

• *Parents love their children.*
• *Farming is hard work.*
• *The child is reading a book.*
• *Children work hard at school.*

Enumerators code whether the woman could: (i) read the full sentence, (ii) read parts of the sentence only, or (iii) not read at all. We consider women who could read the full sentence to be “literate,” as reading one simple sentence is already a low bar for literacy and those who could read “part” of a sentence may have only been able to read as little as a single word.

The FII surveys are nationally representative surveys in ten low- and lower-middle income countries (Bangladesh, Ghana, India, Indonesia, Kenya, Nigeria, Pakistan, Rwanda, Tanzania, and Uganda) and include as respondents both men and women. We use the most recent rounds, collected in 2015, for each country.^8^

The FII surveys ask respondents their highest level of schooling by category and we use the five categories: “no formal education,” “primary education not complete,” “primary education complete,” “some secondary,” and “secondary complete” in our regression analysis. We exclude those who started or completed tertiary, a very small (and highly selected) part of the sampled population.^9^

After completing the main FII questionnaire respondents are asked if they consent to the use of photographs taken by the enumerator in research materials. The respondents are asked to read the three-sentence consent paragraph,^10^ and the enumerator selects the category that corresponds with the respondent’s reading ability: (i) can read the informed consent form fluently without help; (ii) read well but had a little help; (iii) struggled and had a lot of help; or (iv) was unable to read/asked interviewer to read. We define an FII respondent as “literate” if they could read the text without help. The FII administers the literacy test to all respondents and hence does not have the same selection issues as the DHS.

The literacy rates as measured by the DHS and FII in the countries that overlap in the two surveys are similar in levels and strongly correlated across countries for women with similar levels of education (Table 1 in Kaffenberger and Pritchett, 2020a). Thus, while these represent crude measures of literacy, with different assessments and scales, we have some confidence that they are measuring a similar capability of reading a simple text without assistance.

Our category of “literate” is a very low threshold as the OECD defines literacy as “understanding, evaluating, using and engaging with written texts to participate in society, to achieve one's goals, and to develop one's knowledge potential” (OECD, 2009). UNESCO defines literacy as the “ability to read and write with understanding a simple statement related to one's daily life. It involves a continuum of reading and writing skills, and often includes basic arithmetic skills.” As one comparison point, the city of Jakarta, Indonesia participated in the OECD PIAAC (Programme for the International Assessment of Adult Competencies) assessment of adult literacy. In the PIAAC assessment 57% of adults 25–65 with less than upper secondary complete were classified as “below level 1” (the bottom code). In contrast, 77% of those with less than secondary school complete were classified as literate by the FII and 75% of those without secondary education as literate by the DHS. Hence many of those who can read by the DHS or FII criteria have to be in the bottom code of assessed functional literacy by PIACC.

The literacy variables in both the FII and DHS data are categorical (and FII reports only highest level of schooling completed, not years of schooling, and so schooling is also categorical). We use the literacy variables as both a dependent and independent variable in linear regressions, which imposes both cardinality and linearity on a categorical variable. Our checks revealed treating literacy as categorical was a reasonable approximation as goodness of fit did not fall much by imposing linearity as estimates of the move from category to category were roughly the same.

In reporting regression results we re-scale the DHS linear regression schooling coefficients by 6, so the magnitude compares no schooling versus six years complete, roughly equivalent to primary schooling completion. We re-scale the DHS coefficient on literacy by 2, so the magnitude is no literacy versus read without help. The FII schooling coefficient is scaled by 2 to compare no schooling to primary completion and the literacy coefficient by three to represent moving from the bottom to top category in the four category literacy scale. This re-scaling of the raw regression results enables direct comparison of DHS and FII results.

### 2.3 Outcome and co-variates

*DHS outcomes* We analyze three life outcome variables from the DHS: (i) fertility, the woman's self-reported total live births, (ii) child survival rate, the number of living children divided by total number of live births, and (iii) a measure of women's empowerment.

Our measure of women's empowerment is a standard empowerment index (e.g. Kishor and Subaiya, 2008) of the first principal component of the following questions:

QI) (positive indicators) Whether the woman has any say in the following decisions:

• Her own healthcare
• Making large household decisions
• Visiting family or relatives
• What to do with money her husband earns

QII) (negative indicators) Whether the woman believes a husband beating or hitting his wife is justified if the wife:

• Goes out without telling him
• Neglects the children
• Argues with him
• Refuses to have sex with him
• Burns the food

QIII) Whether the woman believes a wife may refuse sex with her husband if he “has other women.”^11^

*Financial behaviors* From the FII surveys, we construct a financial behaviors index as the life outcome of interest. The original objectives of the FII surveys were to measure the uptake and use of financial products and services among the adult population in each country in order to identify potential needs for additional financial services. The surveys thus include several questions on use of services such as bank accounts, mobile money, insurance, and savings instruments as well as questions on financial behaviors such as saving for emergencies, paying bills on time, and planning how to spend money. We construct a principle components index summarizing these financial behavior indicators. We use binary indicators for use of financial services, including bank account use, mobile money account use, and having at least one type of insurance, all of which are common financial inclusion indicators. We also include an ordinal savings variable with values representing not saving, saving with informal financial tools (e.g. saving at home), and saving with formal financial tools (e.g. with a bank or mobile money) to indicate sophistication of savings behaviors. We then include indicators for respondents’ money management behaviors; a binary indicator was included for agreement with each of the following statements:

• “I spend less than I make each month”
• “I have an emergency fund to cover unplanned expenses”
• “I pay my bills on time”
• “My savings are larger than my debts”
• “I am highly satisfied with my present financial condition”

And finally, a categorical variable represented answers to the question, “how often do you make a plan for how to spend your income?” with answer options of “always or most of the time”, “sometimes”, “rarely”, or “never”. The financial behaviors index was standardized to have a mean of zero and a standard deviation of one for each of the 10 surveys.

In all our regression estimates of Eq. (1) we include a set of “control” variables. These are the woman's age (as a cubic), a binary variable for rural/urban residence, a set of variables for the regions within the country and an asset index built using principal components (Filmer and Pritchett, 2001) to proxy for the material status of the household. This implies that, at least in principle, the channels whereby schooling and learning affect life outcomes via higher income/assets are controlled.

### 2.4 Using instrumental variables estimation techniques to correct for measurement error

In Kaffenberger and Pritchett (2020b) we provide estimates of Eq. (1) with a variety of functional forms and estimation techniques. In the present work we focus only on our preferred specification and method. Our preferred estimation technique is to use instrumental variables estimation using enumeration area leave-out-means (EALOM) as instruments. This decision weighs the limitations of a potentially unconvincing instrument versus the dangers of the attenuation bias from measurement error from using OLS. Measurement error is a ubiquitous (and often severe) problem in all of econometrics, and we use IV because our particular situation is a perfect storm of multicollinearity and differential measurement error.

First, schooling and literacy are highly correlated and hence measurement error in either variable strongly affects *both* regression parameter estimates, making one too low (attenuation bias) and the other too high (as a consequence of what we call “partial omitted variable bias”). This makes estimates of the ratio of schooling and learning as causal channels^12^ doubly wrong.

Second, measurement error with correlated variables is a very severe problem when there is *differential* degrees of measurement error, in the sense of the noise to noise plus signal ratio. There are good reasons to believe that assessing whether a person can read one or a few arbitrary sentences or a passage is a very noisy measure of reading, and reading is a very noisy measure of literacy, and even a sophisticated measure of literacy is a very noisy proxy for the variety of learning results that potentially affect life outcomes. While schooling also suffers from measurement error, the measurement error in reading as a proxy for learning that affects life outcomes is likely much larger than errors in self-reported years (or level) of schooling. Differential *relative* measurement error is part of a perfect storm with highly correlated variables as the differential attenuation bias, which likely attenuates literacy coefficients more than schooling, strongly affect OLS estimates of both terms. We regard the OLS estimates, particularly of the relative coefficients of schooling and learning as unreliable.^13^

We use instrumental variables estimation as a technique to correct for measurement error. To create instruments we take advantage of the clustered sampling used by both DHS and FII, in which respondents in the same enumeration area (EA) are geographic neighbors. We create an “enumeration area leave-out-mean” (EALOM) for each individual i, which is the average literacy (or schooling) level of everyone else in the individual's enumeration area j except individual i:

(7) $${\bar{L}}_{i,j}=\sum_{k=1}^{N_{j},k\neq i}L_{i,j}/(N_{j}-1);{\bar{S}}_{i,j}=\sum_{k=1}^{N_{j},k\neq i}S_{i,j}/(N_{j}-1)$$

where $L_{i,j}$ ($S_{i,j}$) is the literacy (schooling) of the $i^{\mathrm{th}}$ woman in the $j^{\mathrm{th}}$ EA and $N_{j}$ is the total number of respondents in enumeration area j.

To produce consistent estimates an instrument must meet two criteria, first stage “inclusion” and structural equation “exclusion”, and there are large literatures on “weak instruments” which demonstrate that the econometric consequences of failing to meet either of these two criteria are severe (Staiger and Stock (1997), Andrews et al. (2019)).

The “inclusion” criteria is that the instrument must be correlated with the variable being instrumented. Weakness in this condition leads to bias, imprecise IV estimates, and incorrect standard errors. A respondent's schooling and literacy levels are plausibly correlated with her sampling cluster neighbors’ as they plausibly had similar opportunities for schooling attendance and may have attended similar quality schools. The F-statistics for inclusion of our EALOMs as instruments are typically above 10, a commonly used threshold for an adequate instrument.^14^ However, as we estimate each survey/round separately, there is substantial variation across countries and we see instances in weak “first stage” instruments producing very imprecise and odd (e.g. wrong signed and excessively large (both positive and negative)) estimates.

The second criteria is that the instrument must satisfy the “exclusion” restriction: the instrument must not have a direct causal impact on the outcome of interest and can therefore be properly excluded from the equation of interest. In this case our exclusion restriction implies that the schooling or literacy of the other women in the enumeration area should not have a direct affect a woman's outcomes. There are at least two ways in which this exclusion restriction could be violated. One is that if there are true “peer effects” in that, say, women learn from having more literate neighbors. Two, there might be enumeration specific factors affecting outcomes, like the quality of available health facilities that are correlated with the instruments. Either of these would cause the exclusion criteria to be violated and hence the IV estimates to not be consistent. As we only have one potential instrument (the “just identified” case) we have no method for testing these alternative hypotheses^15^ and, although the “leave out mean” is not exactly an enumeration area/cluster fixed effect for each woman, they are too close for the data to distinguish.

To be clear, we are not defending the position that our exclusion restriction is exactly and completely true in each country case and that our estimates are therefore (asymptotically) consistent. But the question is not “perfection” versus “nothing” as even if an IV estimate is not consistent the magnitude of the difference between its probability limit and the “true” value is a function of the magnitude of the violation of the exclusion restriction and hence it may well be that, even if not consistent, IV with EALOM is the best alternative. Our position is that we are choosing from a set of available empirical strategies, each of which has its weaknesses. A brief discussion four possible empirical strategies is instructive.

One option is to attempt to find within the DHS data and knowledge of country history a “clean” identifying instrument, like authors that have used the onset of particular policies, like large scale programs of school construction (Duflo, 2001) or free primary schooling (e.g. Osili and Long, 2008; Keats, 2018; Koski et al., 2018), that form a more defensible identifying instrument across cohorts exposed and not exposed to the policy. This present paper has been delayed by a number of years while we (and collaborators) searched for such an instrument at least across a number of countries (including replicating several existing studies) but we have yet to find a sufficiently convincing and feasible instrument of this type.

A second option is to abandon the use of observational data in the DHS altogether and do an actual experiment to produce estimates with more reliable claims to internal validity (conceptually the random allocation of “treatment” and “control” creates a valid instrument to produce consistent estimates). This approach has three serious limitations. First, even an experiment that induced additional years of schooling, say, through a scholarship program (Duflo et al. (2019), Barrera-Osorio et al. (2018)), does not produce consistent estimates of the mediating causal pathways, so does not recover internally valid estimates of the relative schooling versus learning causal pathways (Imai et al., 2010). Second, a true experimental estimate in one, or a small number of countries may not be superior evidence for a given country relative to even an estimate that is inconsistent as there is a trade-off between internal and external validity and relying on the existing experimental evidence to predict causal impact across an array of countries can easily produce estimates across countries with larger root mean square error (RMSE) of prediction than the use of country specific estimates that lack internal validity (Pritchett and Sandefur (2015), Pritchett (2021)). Three, experiments tracking individuals long enough to estimate the connection between quality of learning in primary school and adult outcomes are going to take a long time, be expensive, and hence there are going to be few of them, even in the far future.

Hence the two most relevant options are OLS versus IV with EALOM type (and hence only weakly defensible) instruments. As pointed out by a very helpful and constructive referee, whether IV EALOM is to be preferred to OLS boils down to beliefs about four parameters (or can at least be reduced to such in a simple model): (i) the correlation of “true” schooling and literacy, (ii) the absolute magnitude of noise to signal for schooling, (iii) the absolute magnitude of the noise to signal for the measures of literacy as a proxy for learning, (iv) the magnitude of the impact on the parameter estimates of the violation of the exclusion restrictions for IV EALOM. Our simulation results (not reported) suggested the typical country is in the “perfect storm” region of this four dimensional space: the very high correlation of schooling and literacy and the very high, and differentially high, measurement error of our literacy measures as proxies for learning mean that even pretty large violations of the exclusion restriction still leaves IV EALOM estimates superior in RMSE to OLS. The referee produced their own simulation results in which that was not true and the authors (of course) agree it is true there are regions of the four dimensional parameter space in which OLS is to be preferred. Three points. One, there is no *ex ante* reason to prefer OLS over IV, that is, this decision should not be treated as if OLS is the “default” against which an alternative estimation choice has to be justified, every method has its own strengths and limitations. Two, as indicated above, precisely what is impossible in the “just identified” case is to recover a consistent estimate of the violation of the exclusion restriction so we cannot just plug estimates of the four relevant parameters into an equation and recover which estimation method is best. Three, if in fact the exclusion restriction is violated in a major way, this, in and of itself, has implications for the estimates of the benefits of women's schooling versus learning as this mechanism would depend on there being positive externalities to women's schooling or learning across at least some relevant geographic area. Hence the relevant social return to schooling or learning investments would have to take those into account and hence in the case of large violations of the exclusion restriction *neither* the OLS or the IV as interpreted here produce the conceptually correct estimates for public sector investment decisions.^16^

With that hopefully clear *caveat lector* on estimation methods, let us proceed.

### 2.5 Meta-analysis weighting

We estimate Eq. (1) separately for each survey round using 128 survey rounds for fertility and child mortality, 67 survey rounds for empowerment, and the 10 countries for financial practices. As each regression can be thought of as the empirical result of a separate study we estimate the central tendency for each set of estimates using the random effects meta-analysis formula for the aggregation of the results of different studies, Eq. (8):

(8) $$\beta^{K}=\sum_{i=1}^{N}\frac{\beta_{i}^{K}}{\mathrm{var}\left(\beta_{i}^{K}+\tau^{2}\right)}/\sum_{i}^{N}\frac{1}{\mathrm{var}(\beta_{i}^{K}+\tau^{2})}$$

where $\beta^{K}$ is the weighted sum of betas for either primary schooling, literacy, or basic education (which is a linear combination of schooling and literacy), $\beta_{i}^{K}$ is the coefficient from survey round i, $\mathrm{var}\left(\beta_{i}^{K}\right)$ is the estimate of the variance of $\beta_{i}^{K}$. The $\tau^{2}$ term accounts for the variation between studies (survey/years) in the random effects model. Each estimated coefficient $\beta_{i}^{K}$ is weighted by the inverse of its variance plus $\tau^{2}$, hence more precise estimates are given more weight than less precise estimates (and some IV survey round estimates have very high variance).

We also report the standard error of estimation of this central tendency. As we will see, the standard error of the random effects mean is small relative to the mean, producing very high powered rejections of a null hypothesis that the “typical” effect is zero.

We also report the 20th and 80th percentile of the distribution of the estimates to capture the dispersion of the estimates across countries/survey rounds.

## 3 Empirical results

### 3.1 IV estimates of impact of basic education

**Fig. 3:**
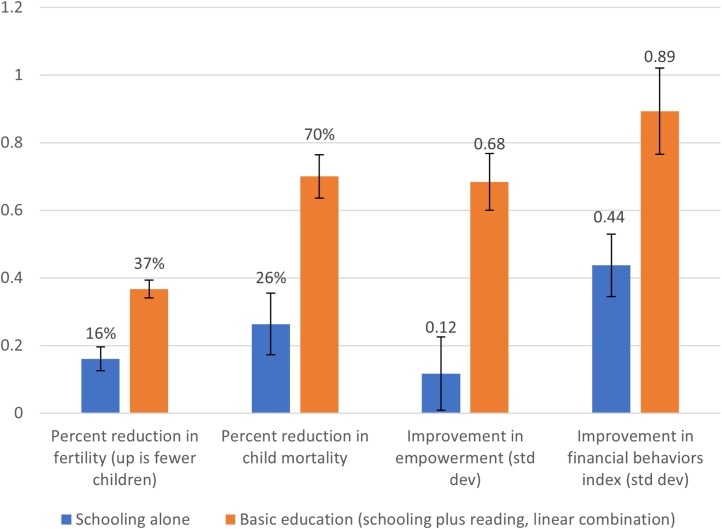
Girls’ basic education, which includes both schooling and literacy, has much larger effects on life outcomes than schooling alone.

Source: Figure shows the random effects weighted average results from IV regressions using 128 rounds of DHS data (fertility, child mortality, and empowerment outcomes) and 10 rounds of FII data (financial behaviors index outcome). "Schooling alone" is the schooling coefficient scaled to represent primary school completion. "Basic education" is the linear combination of the schooling coefficient scaled to represent primary school completion and the literacy coefficient scaled to represent basic literacy.

Table 1 shows the results of estimating Eq. (1) with IV using EALOM as instruments. We focus first on the estimates of basic education, which, given our scaling, is the linear combination of the coefficients of primary schooling and reading. The estimate of the impact of basic education on each of the four life outcomes is practically large and statistically significant. Basic education is associated with a reduction of 1.24 births, from an average of 3.37 (Fig. 3). Basic education is also associated with an increase in child survival of 0.077, which given that child survival in the sample was already 0.89, implies a two thirds reduction in child mortality. Basic education increases the index of female empowerment by 0.684 standard deviations and is associated with an increase in the financial behaviors index of 0.89 standard deviations. The ratios of the mean of the meta-analysis estimate to standard error range from 14 (fertility) to 7 (financial behaviors) which imply the p-levels of the hypothesis test that the mean equals zero would be $10^{-12}$ or smaller.

**Table 1:** IV estimates of primary schooling, reading, and basic education.

	Fertility	Child survival	Women's empowerment	Financial behaviors index
Basic education, linear combination of primary schooling and reading (mean, RE meta- analysis weights)	−1.238	0.077	0.684	0.893
Std. Error of mean coefficient	0.088	0.007	0.084	0.128
Absolute value, ratio mean to std. err.	14.07	11.00	8.14	6.98
20th percentile of distribution of results	−2.542	−0.002	−0.01	0.354
80th percentile of distribution of results	−0.158	0.179	1.922	1.81

Ratio of IV estimate of basic education to OLS estimate of primary schooling (without controlling for reading)	3.76	3.35	4.68	3.20
Ratio of IV estimate of basic education to OLS estimate of basic education	3.32	2.85	3.54	1.78

Schooling (mean, RE meta-analysis weights)	−0.541	0.029	0.117	0.437
Std. Error of mean coefficient	0.121	0.01	0.108	0.093
Absolute value, ratio mean to std. err.	4.47	2.90	1.08	4.70
20th percentile of distribution of results	−2.359	−0.078	−0.804	−0.686
80th percentile of distribution of results	0.967	0.154	1.451	0.758

Reading (mean, RE meta-analysis weights)	−0.566	0.032	0.538	0.368
Std. Error of mean coefficient	0.119	0.011	0.128	0.158
Absolute value, ratio mean to std. err.	4.76	2.91	4.20	2.33
20th percentile of distribution of results	−2.27	−0.078	−1.004	−0.396
80th percentile of distribution of results	0.905	0.22	1.525	2.416

Ratio of reading coefficient to primary schooling	1.05	1.10	4.60	0.84

Number of survey rounds	128	128	67	10

Note: Regressions contain controls for age, age squared, age cubed, asset index, a rural/urban dummy, and dummies for regions. Schooling coefficients have been scaled to reflect primary school (six years) completion; reading coefficients are scaled to reflect going from no reading to reading without help.

**Fig. 4:**
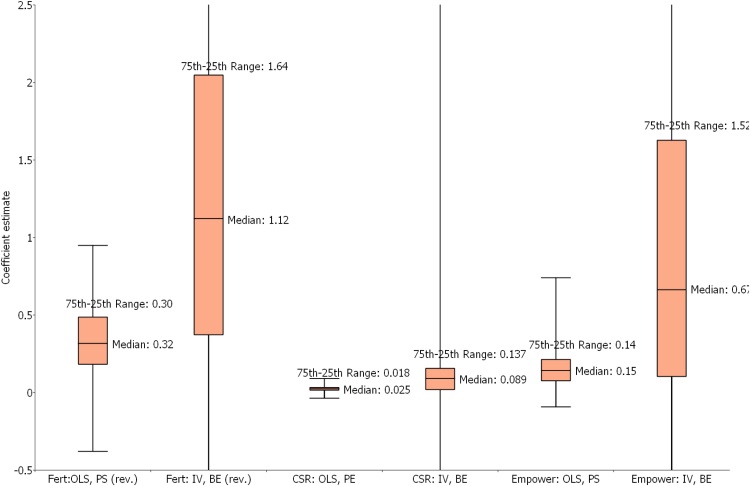
Comparing the distribution of estimates of primary schooling with OLS versus basic education using IV.

Source: Figure shows the median, interquartile range, and minimum and maximum coefficient values for primary school, estimated using OLS, and for basic education (linear combination of primary school and literacy) using IV, across 128 DHS survey rounds for fertility and child survival and 67 DHS survey rounds for empowerment.

The reported 20${}^{\mathrm{th}}$-80${}^{\mathrm{th}}$ percentiles of the distribution across countries of the estimated impact of basic education shows considerable variability. In the 80${}^{\mathrm{th}}$ percentile (high impact) countries the estimates are typically more than twice as large as the estimated mean. For example, the mean impact of basic education on women's empowerment is 0.68 but in the 80${}^{\mathrm{th}}$ percentile country the estimate is 1.9, the mean impact on child surival is 0.077 but the estimate is 0.18 in the 80${}^{\mathrm{th}}$ percentile country. Conversely, the estimates in the 20${}^{\mathrm{th}}$ percentile country are quite low, and child survival and women's empowerment the 20${}^{\mathrm{th}}$ percentile estimate is of the “wrong” sign.^17^ This large heterogeneity across countries is in some part due to the large imprecision in the individual countries estimates induced by the IV estimation technique (shown in Fig. 4), but is also certainly due in some part to underlying differences across countries in the “true” impact of education on outcomes, as, given the very large differences across countries in economic and social conditions there is no reason to expect the impact of basic education would be the same.

Fig. 4 and row 6 of Table 1 show that IV estimates of the impact of basic education are much larger than the standard practice of using OLS regressions of outcomes on schooling. Our estimates of the average impact are 3.2 (financial behaviors) to 4.7 (women's empowerment) larger than the OLS estimates of the impact of primary schooling (detailed OLS estimates are reported in Kaffenberger & Pritchett (2020b)). For instance, the the average of the OLS estimates of the impact of primary schooling on women's empowerment is 0.146 versus 0.684 with IV (of an index with standard deviation equal to 1). The box plots of the estimates from the DHS outcomes show that the median IV estimate of the impact of basic education on fertility is a reduction of 1.12 births whereas the median of the OLS estimates of the impact of primary schooling is a reduction of 0.32 births. Fig. 4 shows that, even though the increased imprecision from IV estimates makes for a large dispersion in the estimates, the 25${}^{\mathrm{th}}$ percentile of the IV estimates is as high or higher than the median OLS estimate.

An important point about method is that the principal reason our IV results are larger is *not* that we include an explicit variable of learning (in this case reading) but rather because of *both* allowing for schooling and learning to have separate channels of influence *and* the use of IV estimates to account for measurement error. Comparing Rows 6 and 7 of Table 1 shows that most of the difference is between IV and OLS estimates of the impact of basic education.

Table 1 also provides estimates of the coefficients of schooling and reading separately. These results show that the combination of the use of IV with EALOM as instruments and the high correlation of schooling and learning leads to very imprecise estimates of the individual components. For instance, the 20${}^{\mathrm{th}}$-80${}^{\mathrm{th}}$ range of the estimate of primary schooling (conditional on reading) runs from reducing fertility by 2.5 births to raising it by 1 birth, and the impact of reading (conditional on schooling) similarly runs from a 2.3 birth reduction to a 0.9 birth increase. This is primarily due to the very large standard errors of the estimates for each country, which produces at times wildly implausible results (even when the estimate of the sum of the two coefficients (basic education) is quite precise).

The average of the IV estimates of primary schooling and reading are about equal for three of the outcomes (fertility, child survival, and financial behaviors) while the average of the estimates for reading is much larger for women's empowerment (0.538 vs. 0.117) (Fig. 3). This suggests that neither extreme view: that all of the impact of education is transmitted just by schooling or that all of the impact is due to learning and the direct impact of schooling (“time served”) is zero, is supported by the present results.

Table 2 provides a thought experiment to illustrate the implications of differential production of reading during primary school across countries. The table uses the RE weighted average estimated coefficients of the direct impact of primary schooling and of achieving reading from Table 1 to calculate the difference in the total impact of schooling (Eq. (3)) across countries with different levels of the production of reading from primary school $\gamma^{c}$ (as shown in Fig. 1). Egypt is (roughly) the 20${}^{\mathrm{th}}$ percentile country and six years of schooling produce an increment to reading (on a 0 to 2 scale) of 0.95 while Peru is (roughly) the 80th${}^{\mathrm{th}}$ percentile country and six years of schooling in Peru produces a reading gain of 1.73.^18^ If we assumed that $\beta_{S|L,Z}^{c}$ and $\beta_{L|S,Z}^{c}$ are the same in the two countries, at the overall country RE weighted average (which we know they are not, but for this thought experiment we will assume they are), the total impact of primary schooling in Peru is going to between between 37 percent (financial behaviors) and 67 percent higher (women's empowerment) because women learn more.

(9) $$\mathrm{Ratio}\mathrm{of}\mathrm{total}\mathrm{impact}\mathrm{of}\mathrm{schooling},A\mathrm{vs}.B,\mathrm{same}\mathrm{coefficients}=\frac{\beta_{S|L,Z}+\gamma^{A}*\beta_{L|S,Z}}{\beta_{S|L,Z}+\gamma^{B}*\beta_{L|S,Z}}$$

**Table 2:** Differential learning leads to differential predicted impact on women's life outcomes from primary schooling

Impact of schooling	Gain in reading (0 to 2 scale) from six years of schooling)	Fertility	Child survival	Women's empowerment	Financial behaviors
Egypt (20th percentile)	0.95	−1.08	0.060	0.63	0.79
Peru (80th percentile)	1.73	−1.52	0.085	1.05	1.08
Extra impact from higher level of reading acquisition from primary schooling		40.9%	42.0%	66.7%	36.5%

Nigeria	0.66	−0.91	0.050	0.47	0.68
Rwanda	1.68	−1.49	0.083	1.02	1.06
Extra impact from higher level of reading acquisition from primary schooling		63.6%	65.6%	117.2%	55.6%

Average in DHS rounds	1.38	−1.32	0.07	0.86	0.94
Universal reading (all reach 2)	2	−1.67	0.093	1.19	1.17
Extra impact from higher level of reading acquisition from primary schooling		26.5%	27.1%	38.8%	24.1%

Note: Table shows the hypothetical impact of schooling on each outcome at the random effects weighted average coefficient for schooling and reading and the country-specific γ, or production of reading per year of schooling. These calculations use the DHS and FII estimates of the gain to reading from six years of schooling, averaged across survey rounds, and the random effects weighted average coefficients for schooling and reading on each life outcome reported in Table 1.

The same calculation is shown comparing the impact of schooling on outcomes at the average level of learning, $\gamma^{\mathrm{Avg}}=1.38$, versus if reading were universally acquired through primary schooling. If countries were able to accomplish universal literacy during the primary years, the positive impact on women's life outcomes from completing primary schooling would improve between 24 percent (financial behaviors) and 39 percent (women's empowerment).

### 3.2 Caveats

Before examining the implications of these estimates for cost benefit analysis for policy and program design in Section 4, we would like to point out some methodological issues and highlight three major limitations of our results.

An initial issue to clarify is why, when the field of development is smitten with RCTs as a method to produce unbiased estimates of the causal impact, or LATEs, of various actions (policies/programs/projects), one would even bother with estimates from observational data. Four quick points on why RCTs don’t (won’t) resolve the questions we are addressing. First, even if an RCT shows an action increases schooling and that increase thereby improves life outcomes, to the extent that any part of the causal pathway is through learning ($\beta_{L|S,Z}^{c}>0$) this LATE has no external validity as the impact of schooling depends on the extent of learning (Table 2 and we know this varies widely across contexts (Fig. 1)). A “rigorous” estimate of the impact of schooling from a low learning country could dramatically understate the impact of schooling in a different country and the impact of women's education (which includes schooling and learning). Second, as the example of Barrera-Osorio et al. (2018) in Section 2 shows, RCT estimates of the LATE of actions to increase grade attainment do not reveal life impacts, as the intervention increased schooling for both treatment groups but learning and life outcomes for only those in the group selected by merit. Those not selected through merit may have needed more learning-focused efforts to achieve improvements to learning and life outcomes. A review article, Ganimian and Murnane (2016), found that in the literature on cash inducements to increase schooling, including scholarships and conditional cash transfers, nearly all had positive impacts on schooling attainment, but only merit-based programs, meaning those who selected children with higher test scores, had positive impact on learning achievement. One cannot assume that the impact on life outcomes of an incremental grade attainment is invariant to how, and for who, the grade attainment was increased. For instance, there is very strong evidence that conditional cash transfers (CCTs) increase school attendance (Attanasio et al., 2012, Behrman et al., 2005). However, if CCTs induce children to return to schools from which children had dropped out because they were not learning (Kaffenberger et al., 2021) and hence this increased grade attainment induces little additional learning (Millán et al., 2019, Behrman et al., 2008), then the impact on life outcomes will be less than the “average” impact of schooling on life outcomes. In such situations, learning focused interventions may be needed.

Third, even if an RCT tracks the impact of an intervention on schooling and on learning through schooling and also impact on life outcomes, this does not, in general, produce an unbiased estimate of learning on life outcomes, for the same selectivity problems discussed below. Fourth, as our results are at least suggestive of substantial heterogeneity in the impact of education across contexts, even if one had one or a handful of RCT estimates of the impact of education on life outcomes (and not just grade attainment) it cannot be taken for granted this would help predict the impact of education in any other context Pritchett (2021). So, while RCTs have their uses, they cannot resolve all the issues needed for evidence based decisions about schooling, learning, and improving life outcomes.

Some caveats about our estimates.

First, the imprecision of the IV estimates of the schooling and reading coefficients becomes very large in many survey rounds and the coefficients take on implausible values. However, since the coefficient estimates are so highly correlated, when one of the coefficient estimates (schooling or learning) is wildly implausible (e.g. large and of the wrong sign) that is typically compensated in the linear combination of the impact of education by the other coefficient being implausibly large in the opposite direction. Moreover, the co-variance between the estimates reduces the standard error estimate for the linear combination. Hence, implausible and imprecise estimates of each term add up to a plausible magnitude and reasonably precise estimate of the impact of basic education. However, the decomposition into schooling and learning is plausible only in the aggregation, not case by case.^19^

Although we have been explicit that the word “impact” is used for convenience and not as a claim or as an interpretation of our estimates, in our case there are both the standard concerns about the bias induced by “selectivity” and an issue specific to our study.

The standard issue is that one cannot treat the differences in years of schooling and learning across women as if these were randomly assigned as they are the result of choices (under constraints) made by women (and their parents) when they were young. This raises the plausible objection that women who completed more schooling had other characteristics, not included in our regressions, which had a direct impact on life outcomes and hence the estimated impacts are likely biased upward. There are three considerations. One, our empirical results are no worse in this regard than nearly all of the existing literature estimating the returns to women's schooling.^20^ Two, by examining non-pecuniary life outcomes (not money wages in employment) we mitigate the implications of schooling producing higher wages via signaling (Michael, 1973, Caplan, 2018), as there is no “third party” employer to whom more schooling is a signal. Third, (as alluded to above) the fact that the DHS sample is censored above–only those with primary school as their highest level are included in the DHS sample-implies that we are only comparing outcomes among those women who did not choose (or were not able) to attend secondary school or higher. The usual concern in this literature is that if one compares life outcomes for women with secondary schooling to those without secondary schooling in a setting where secondary schooling completion is rare this raises the possibility that women with secondary school are strongly selected on ability or grit or unobserved positive background characteristics that directly affect results. However, given the DHS decision to not assess literacy of women with any secondary schooling these women are not in our samples.

The econometric issue specific and important to our paper is the decomposition of pathways into schooling and learning. The intuitive answer to “how bad is the bias from lack of random assignment?” depends on how much of the variation in the independent variables in the data is “as if” it were due to random assignment in that whatever determined the value of the independent variable was not correlated with the outcome. One could imagine that historically lots of people lived in rural areas, schools were relatively rare, and what schools there were were “as if” randomly placed relative to the characteristics of the people who attended them. In such a scenario, whether or not the adult women we observe in the DHS have no schooling or have primary schooling might heavily depend on whether there was a (somewhat randomly placed) school nearby when they were young. In this case the bias from selectivity, that women who had primary schooling also have characteristics more likely to make them have good outcomes, might be modest.^21^ However, in order to identify the literacy (or more generally, the learning) impacts one needs variation in the amount of measured learning of individuals with the same degree of schooling. While some of that variation may be “as if” randomly assigned because some children were proximate to good (high value added) schools and others happened to be proximate to bad (low value added) schools, the evidence is pretty powerful that far and away the most powerful correlates with measured learning are child background characteristics (like SES). And, it is quite easy to believe that the same individual characteristics that account for higher learning, conditional on attending a given level of schooling are those characteristics that lead to more favorable life outcomes. The upshot of this is that, even if schools are only more randomly assigned *relative to* learning, then there are reasons to believe the bias in OLS (or IV) coefficients relative to a LATE are larger for learning than for schooling.

## 4 Illustration of costs and benefits of increasing schooling grade attainment versus raising learning: Implications for investing in education

Whether our particular estimates are correct or not, decisions about how to invest in education to improve pecuniary (wages, incomes) or non-pecuniary (child mortality, empowerment) life outcomes *necessarily* depend on an understanding of the channels whereby schooling has its impact on outcomes. There is a massive, and rapidly expanding, literature creating estimates of the impact and cost-effectiveness of various interventions using rigorous methods for estimating causal impacts of interventions on schooling or on learning per year of schooling.^22^ Without estimates of the *relative* impacts of schooling (grade completion) versus learning (on some measure) on life outcomes the estimates commonly produced of the impact of and cost effectiveness of various interventions in either raising schooling completed (at existing learning per year) or raising learning are an inadequate guide to education investments.^23^

Suppose there was an intervention (program, project, policy) that at marginal cost $c^{S}$ could raise girl's schooling by one year (we set $c^{S}$ to equal 1 as a normalization) and another intervention that at marginal cost $c^{\gamma }$ could raise the learning per year of schooling, γ. Which is the most cost effective investment to increase the impact of education on a life outcome (child mortality, wages, empowerment, etc.)? The standard optimizing decision rule is equate the marginal benefit per dollar across the two possible interventions.

(10) $$\left(\frac{\mathrm{MB}}{\mathrm{MC}}\right)\;{}^{\gamma }=\left(\frac{\beta_{L|S,Z}*S}{C^{\gamma }}\right)$$

(11) $$\left(\frac{\mathrm{MB}}{\mathrm{MC}}\right)\;{}^{S}=\left(\frac{\beta_{S|L,Z}+\beta_{L|S,Z}*\gamma }{C^{S}(\equiv 1)}\right)$$

Combining Eqs. (10) and (11) imply that the cost of a learning-increasing intervention (relative to the cost of an incremental year of schooling which is normalized to 1) that would equalize the MB per dollar of the two interventions in producing a particular outcome Y is:

(12) $${\mathrm{MC}}^{*}(\gamma )=\frac{\beta_{L|S,Z}*S}{\beta_{S|L,Z}+\beta_{L|S,Z}*\gamma }$$

Eq. (12) is in terms of scaled quantities (the β are not scaleless elasticities but are derivatives in units specific to the particular outcome, e.g. fewer children per year of schooling) and so cannot be interpreted directly, but the implications of Eq. (12) are quite intuitive.

One, if none of the causal impact of schooling is through a measure of learning L ($\beta_{L|S,Z}=0$) then it can never be worth investing in the learning-increasing intervention. To some extent this is the implicit assumption behind maximizing school attendance and grade attainment without any attention to learning.

Two, if none of the causal impact of schooling is the “direct” effect of schooling ($\beta_{S|L,Z}=0$) then ${\mathrm{MC}}^{*}(\gamma )=\frac{S}{\gamma }$. This has the simple and clear implication that the higher S the more gain to increasing learning per year. And, the lower the existing level of learning γ the higher the return to investing in improving learning.

Three, when both the “direct” impact of schooling and learning are positive, the highest that the marginal cost a learning increasing intervention can be and still be optimal depends on the magnitudes of both coefficients. In Table 3 we illustrate the implications using our IV estimates. The first column is the “base case” where we assume a country has six years of schooling and the gain in reading (on 0 to 2 scale) per year of schooling is 0.22. In this case the optimal marginal cost for actions (policies/programs/projects) that increase learning relative to the cost of an additional year of schooling is 13, one could spend 13 times as much to improve learning per year in each grade and still be cost effective in improving life outcomes. The differentials across the columns are also instructive.

**Table 3:** The highest optimal cost of increasing learning relative to schooling (cost of achieving one additional year set to 1) given the estimated pathways to outcomes.

Illustrative case	Base case	Low learning	High learning	Low schooling	High schooling
Schooling years	6	6	6	3	9
Reading gain per year of schoolin (on 0 to 2 scale)	0.22	0.11	0.33	0.22	0.22

Fertility	11.1	14.0	9.3	5.6	16.7
Child survival	11.5	14.5	9.5	5.7	17.2
Empowerment	20.5	32.9	14.9	10.3	30.8
Financial behavior	9.7	11.9	8.3	4.9	14.6
Average of the four life outcomes	13.2	18.3	10.5	6.6	19.8

Note: This is Eq. (12) using unscaled coefficients from Table 1. The cost of increasing school attainment by one year is normalized to 1.

One, the lower the current level of learning being produced by a year of schooling (γ) the higher the relative costs that would be optimal to incur to improve learning. With low learning gain of 0.11 the average across the four life outcome optimal marginal cost is 18.3 (versus 13.2 in the base case) whereas when learning is high (0.33) the gain is 10.5

Two, the relative benefit of improving learning is higher when the level of schooling is higher. When S=9 the ratio is 19.8 (versus 13.2) whereas when S=3 (column 8) the ratio is only 6.6. This is intuitive as the higher learning per year applies to more years of schooling. Countries that have already achieved relatively high levels of schooling attainment but at low levels of learning could vastly increase life benefits by increasing the learning per year from their schooling.

Third, the relative benefits of investing in learning are higher the larger the relative channel of impact on outcomes is through learning versus the “direct” effect of schooling. This can be seen in two ways. As seen in Table 1 the relative impact of learning to schooling is higher for empowerment (0.538 versus 0.117) than for financial behaviors (0.368 versus 0.467) and hence (in Table 3) at S=6 and γ=.22 the relative benefit of learning to schooling is 20.5 for empowerment but only 9.7 for financial behaviors.

## 5 Conclusion

The education of girls has rightly received enormous attention and is widely regarded as a critical development investment for countries. However, the conflation of “schooling”–measured as just “time served” in a building called a school–and “education”–the acquisition of skills, competencies, and capabilities, leads to confusion. We show that in producing life outcome benefits for adult women via education that both the duration of schooling and learning (proxied by reading) matter. This leads to two main points.

One, using our estimation techniques and data that allow us to incorporate the benefits of learning (reading) we find that the impact of *basic education*, defined as completing primary school and learning to read, has three times larger impact on four different life outcomes than the usual estimation techniques for women's schooling would suggest. While there are powerful caveats and our results are far from the final word, the evidence is at least suggestive that women's education is much better than the existing evidence suggests.

Two, deciding on the allocation of spending and effort across increasing schooling grade attainment versus raising learning necessarily depends on assumptions about the causal drivers of improved life outcomes. In current discussions these assumptions are often implicit or *ad hoc*. Our estimates suggest that efforts to produce higher learning outcomes for girls already attending schools could be *orders of magnitude* more cost effective in producing improved life outcomes than spending to extend schooling at existing levels of learning.

## Author statement

**Michelle Kaffenberger:** Conceptualization, Methodology, Formal analysis, Writing – original draft, Writing – Review & editing.

**Lant Pritchett:** Conceptualization, Methodology, Formal analysis, Writing – original draft, Writing – Review & editing.

## Footnotes

1 This paper is a radically shortened version of an earlier working paper (Kaffenberger and Pritchett, 2020b) which runs to 80 pages with 8 appendices that has much more technical exposition and detailed comparison of various empirical results. This work in turn drew on Oye et al. (2016). We would like to thank Justin Sandefur for close collaboration and discussions over the years that led to this paper, his inputs and outputs have been critical to improving this work to where it is, but he is not implicated in its remaining weaknesses. We would also like to thank participants in a RISE workshop where it was presented for the suggestions, Deon Filmer for support on an earlier version of this work, and Clare Leaver for helpful comments and feedback.

2 A big caveat on our use of terminology: we use the word “impact” to refer to the empirical association (either partial or total derivative) estimated from a multivariate regression framework, not because we (naively) believe observational data produces unbiased/consistent estimates of a causal impact or LATE (local average treatment effect), but because other circumlocutions for “impact” are so awkward and unwieldy. If the reader (reviewer/referee) wants to mentally search and replace our use of “impact” with “partial (or total, depending on context) derivative of y with respect to x as estimated from linear (though this linearity is inessential) multivariate regression using observational data” nothing about our argument will be affected. We try and use notation and language (and periodically repeat caveats) that make clear our use of the word “impact” is short-hand for a particular coefficient, from a particular model, estimated in a particular way, not an assertion of identification of the “true” causal impact.

3 Note that the interpretation of “can read with some help” will vary across countries, languages, and scripts; we use “can read with no help” as our definition of basic literacy, as discussed in Section 2.

4 This “total” ignores the effect of schooling and learning on the other factors, Z, for instance, if higher schooling raised incomes/wealth, and so this is really a “partial total” but we ignore those other pathways for simpler exposition.

5 The emphasis on *all* is to stress that this is not the result of some flaw in estimation technique but that even the *correctly* estimated (e.g. unbiased) estimate of the LATE of an incremental year of schooling on outcomes depends on the learning from the year of schooling and whether a causal pathway of schooling on outcomes is through learning and hence the actual, correctly estimated, impact of schooling underestimates the gains that would have been achieved from education–schooling plus learning.

6 Even well identified causal estimates of the impact of school cannot overcome these as identifying the channels through which schooling impacts outcomes requires estimates of $\gamma^{c}$ and $\beta_{L|S,Z}$. For instance, Breierova and Duflo (2004) exploit variation from a nationwide school construction program in Indonesia to recover causal estimates of the impact of increased parental schooling on child mortality. Similarly, introduction of Universal Primary Education in Nigeria in 1976 and Uganda in 1997 provided researchers with a source of exogenous change; based on this analysis Osili and Long (2008) suggests that increasing female schooling by one year reduces early fertility in Nigeria, and Keats (2018) finds that women in Uganda with more schooling prefer to have fewer children, delay having their first child, and reduce overall fertility at any age, while investing more in their children's health. But, no matter how well identified or precise, the estimates of the total impact of school, even cleanly identified causal estimates, do not provide any information on the impact of *learning* ($\beta_{L|S,Z}$) and hence of education - or what could be achieved if schooling produced learning. Moreover, even if a randomized or experimental design produces clean identification of the impact of schooling, and even if the study includes data on learning (e.g. literacy) this still would not provide well-identified estimates of the causal pathways ($\gamma^{c}$.) Further, such estimates of the causal impact of schooling on mortality in Indonesia depends on $\gamma^{\mathrm{Indonesia}}$ and hence, even if $\beta_{S|L,Z}$ and $\beta_{L|S,Z}$ themselves were constant across all countries the estimate from Indonesia cannot be used to estimate the impact of schooling in countries with much lower (e.g. in the DHS data for instance, Ghana, Nigeria) or much higher learning countries unless $\beta_{L|S,Z}$ is zero.

7 The data report those for whom an appropriate language card was not available and this was typically a quite small percent. These women, by not having a literacy result also do not figure in our results.

8 More information on the surveys can be found here: finclusion.org

9 The main econometric concern is the combination of the possibility there is a non-linear relationship between measured schooling and outcomes and that tertiary education is highly selective and therefore using a linear regression might cause the estimates to be leveraged up by this part of the sample and hence the linear estimates would not actually be a good estimate of the incremental benefit of moving from, say, 6th to 8th grade. We could have kept these observations in the sample and then allowed for non-linearity (e.g. allowed for splines in the impact terms) and focused on our range of interest, but it is simpler to just drop these observations.

10 The exact English text from the Kenyan survey instrument is: “We would like to take some photographs of you and your household. We will include some of the photographs in our reports. We might also publish some of them online on our website.” This text was translated into the relevant local languages.

11 The empowerment index is estimated separately for each survey round and is normalized within each survey round to mean of zero, standard deviation 1. Hence the coefficients are comparable across countries in standard deviation units but these may represent different “absolute” amounts.

12 Which feeds into many formula, like the relative MB to MC of learning vs. schooling in Eq. (12) in Section 4.

13 In Kaffenberger and Pritchett (2020b), Appendix E provides a (mildly) technical primer on the consequences of differential measurement error with correlated variables and Appendix H provides estimates of OLS using the woman level data versus regional or enumeration area averages, as these three levels of aggregation provide some indications of the severity of measurement error.

14 Stock and Yogo (2005) show that this threshold is not accurate and depends on a number of aspects of the problem. For a single variable to be instrumented and a single instrument the critical values range from 5.5 to 16.4 depending on the desired maximal size of a 5 percent Wald test (their Table 5.2).

15 Kaffenberger and Pritchett (2020b) discusses more fully the limitations of IV with EALOM in Appendices F and G.

16 A classic illustration of the economics of interventions with local (geographically limited) externalities is deworming where the social benefits exceed the private benefits and justify a sustained subsidy ((Kremer and Miguel, 2007))

17 Although across all four indicators (345 distinct estimates) there is only one that is both “wrong” sign and significant.

18 This is “roughly” because these countries have multiple DHS survey rounds (we have 128 survey rounds across 54 different countries) and in the calculations in Table 2 we use the country average across all rounds. Reassuringly, these estimates are very stable across survey rounds.

19 This is explicated and illustrated with graphs for the three DHS outcomes in Appendix F and Appendix G of Kaffenberger and Pritchett (2020b)

20 Mensch et al. (2019) for instance, find in their systematic review only 16 studies on the question of women's education and child and maternal health that pass their filters for causal identification.

21 One could argue that the most striking thing about the famous Duflo (2001) use of the rapid expansion of schools in Indonesia in the 1970s as an exogenous “natural experiment” to instrument for schooling attainment to estimate wage returns to schooling is how very much like the simplest OLS estimate the sophisticated IV estimate is.

22 See, for example: Dhaliwal et al., 2013, Ganimian and Murnane, 2016, Glewwe and Muralidharan, 2016i, though overall cost-effectiveness as part of impact evaluation is still not common (Brown and Tanner, 2019).

23 This is in no way an argument against schooling for all children - all children everywhere have the fundamental right to an education. It is rather an acknowledgement that, for children who are in school, there may be tradeoffs between prioritizing further school expansion (such as universal secondary completion) and learning for all, and the consequences for life outcomes of each are needed to inform the decision making process.

## References

[bib0005] Attanasio Orazio P., Meghir Costas, Santiago Ana (2012). Education choices in Mexico: using a structural model and a randomized experiment to evaluate progresa. Rev. Econ. Stud..

[bib0010] Andrews Isiah, Stock James, Sun L. (2019). Weak instruments in iv regression: theory and practice. Ann. Rev. Econ..

[bib0015] Barrera-Osorio Felipe, de Barros Andreas, Filmer Deon (2018). World Bank Policy Research Working Paper, No. 8566.

[bib0020] Behrman Jere, Sengupta Piyali, Todd Petra (2005). Progressing through progresa: an impact assessment of a school subsidy experiment in rural mexico. EDCC.

[bib0025] Behrman Jere, Parker Susan W., Petra Todd (2008).

[bib0030] Breierova Lucia, Duflo Esther (2004). The impact of education on fertility and child mortality: do fathers really matter less than mothers?. NBER.

[bib0035] Brown Elizabeth Denison, Tanner Jeffery (2019). World Bank Policy Research Working Paper, WPS 9041.

[bib0040] Caplan Bryan (2018).

[bib0045] Cleland J.G., Van Ginneken J.K. (1988). Maternal education and child survival in developing countries: the search for pathways of influence. Soc. Sci. Med.(1982).

[bib0050] Cochrane Sarah (1980). World Bank Sector Working Paper, SWP405:1.

[bib0055] Dhaliwal Iqbal, Duflo Esther, Glennerster Rachel, Tulloch Caitlin (2013 12). http://hdl.handle.net/1721.1/116111.

[bib0060] Duflo Esther, Dupas Pascaline, Kremer Michael (2019).

[bib0065] Duflo Esther (2001). Schooling and labor market consequences of school construction in indonesia: evidence from an unusual policy experiment. Am. Econ. Rev..

[bib0070] Filmer Deon, Pritchett Lant H. (2001). Estimating wealth effects without expenditure data-or tears: an application to educational enrollments in states of India. Demography.

[bib0075] Gakidou Emmanuela, Cowling Krycia, Lozano Rafael, Murray Christopher J.L. (2010). Increased educational attainment and its effect on child mortality in 175 countries between 1970 and 2009: a systematic analysis. Lancet (London, England).

[bib0080] Ganimian Alejandro J., Murnane Richard J. (2016). Improving education in developing countries: lessons from rigorous impact evaluations. Rev. Educ. Res..

[bib0085] Glewwe Paul, Muralidharan Karthik (2016). https://ideas.repec.org/h/eee/educhp/v5y2016icp653-743.html.

[bib0090] Imai Kosuke, Keele Luke, Yamamoto Teppei (2010). Identification, inference and sensitivity analysis for causal mediation effects. Stat. Sci..

[bib0095] Kaffenberger Michelle, Pritchett Lant (2020). Aiming higher: Learning profiles and gender equality in 10 low- and middle-income countries. Int. J. Educ. Dev..

[bib0100] Kaffenberger Michelle, Pritchett Lant (2020). RISE Working Paper Series, 20/049.

[bib0105] Kaffenberger M., Sobol D., Spindelman D. (2021). The role of low learning in driving dropout: A longitudinal mixed methods study in four countries. RISE Working Paper 21/070.

[bib0110] Keats Emily C., Ngugi Anthony, Macharia William, Akseer Nadia, Khaemba Emma Nelima, Bhatti Zaid, Rizvi Arjumand, Tole John, Bhutta Zulfiqar A. (2017). Progress and priorities for reproductive, maternal, newborn, and child health in Kenya: a Countdown to 2015 country case study. Lancet Glob. Health..

[bib0115] Keats Anthony (2018). Women’s schooling, fertility, and child health outcomes: evidence from Uganda’s free primary education program. J. Dev. Econ..

[bib0120] Kishor Sunita, Subaiya Lekha (2008). DHS Comparative Reports No. 20.

[bib0125] Koski A., Strumpf E.C., Kaufman J.S., Frank J., Heymann J., Nandi A. (2018). The impact of eliminating primary school tuition fees on child marriage in sub-saharan africa: a quasi-experimental evaluation of policy changes in 8 countries. PLoS One.

[bib0130] Kremer Michael, Miguel Edward (2007). The illusion of sustainability. Q. J. Econ..

[bib0135] Martin Teresa Castro (1995). Women’s education and fertility: results from 26 demographic and health surveys. Stud. Fam. Plan..

[bib0140] Mensch Barbara S., Chuang Erica K., Melnikas Andrea J., Psaki Stephanie R. (2019). Evidence for causal links between education and maternal and child health: systematic review. Trop. Med. Int. Health.

[bib0145] Spence Michael (1973). Job market signaling. Q. J. Econ..

[bib0150] Millán Teresa Molina, Barham Tania, Macours Karen, Maluccio John A., Stampini Marco (2019). Long-term impacts of conditional cash transfers: review of the evidence. World Bank Res. Obser..

[bib0155] Montenegro Claudio, Patrinos Harry Anthony (2014). World Bank Policy Research Working Paper, 7020.

[bib0160] OECD (2009). OECD Education Working Papers, 34.

[bib0165] Osili Una Okonkwo, Long Bridget Terry (2008). Does female schooling reduce fertility? Evidence from Nigeria. J. Dev. Econ..

[bib0170] Oye Mari, Pritchett Lant, Sandefur Justin (2016). The Education Commission Background Paper.

[bib0175] Pritchett Lant (2013).

[bib0180] Pritchett Lant, Sandefur Justin (2015). Learning from experiments when context matters. Am. Econ. Rev..

[bib0185] Pritchett Lant (2021). http://lantpritchett.org.

[bib0190] Psaki Stephanie R., Chuang Erica K., Melnikas Andrea J., Wilson David B., Mensch Barbara S. (2019). Causal effects of education on sexual and reproductive health in low and middle-income countries: a systematic review and meta-analysis. SSM - Popul. Health.

[bib0195] Staiger Douglas, Stock James H. (1997 May). Instrumental variables regression with weak instruments. Econometrica.

[bib0200] Stock James, Yogo Motohiro (2005).

[bib0205] United Nations (1985).

